# Diseases of the Nervous System

**Published:** 1905-05-06

**Authors:** 


### DISEASES OF THE NERVOUS SYSTEM.

**The Cerebro-Spinal Fluid** is the subject of an elaborate lecture by Mott,[1] who said that it has chemical and physical properties different from those of any other fluid in the organism. It is clear, with a specific gravity of 1·006, slightly alkaline, and devoid of all corpuscular elements. Its principal constituents are sodium chloride with traces of carbonates, phosphates, urea, dextrose, and globulin. It does not contain either albumen or fibrinogen. Its average amount is from 120 to 150 c.cm. It is continually being secreted by the epithelium of the choroid plexuses of the lateral ventricles, and as continually escapes through the foramen of Majendie into the subarachnoid space, and passes from the cranium to the spinal canal at each cardiac systole. It is not exactly determined how it escapes, but probably along the lymphatics of the perineural sheaths of all the cranial and spinal nerves, and arriving thus at the thoracic duct. It also, according to Cushing, escapes from the cerebral subarachnoid space and perivascular canalicular systems into the longitudinal sinus. The cerebro-spinal fluid thus fills up all the spaces in the cranium and spinal canal which are not occupied by tissues or blood, equalises the pressure throughout and acts as a water-cushion, especially at the base of the brain. The metabolism of the brain is very small, and the products of activity are continually being neutralised and removed by the cerebro-spinal fluid. Interesting pathological support for the view that the cerebro-spinal fluid is secreted by the choroid plexuses is found in cases of tumour blocking the iter. These cause internal hydrocephalus—distension of the lateral ventricles and third ventrical—and, clinically, signs of increased intracranial pressure—vomiting, headache, optic neuritis, tremors, fits, and drowsy stupor, with progressive mental enfeeblement. The composition of the cerebro-spinal fluid becomes altered in disease, *e.g.* in uræmia urea is present in appreciable quantities, in diabetes the sugar is increased, but in jaundice the fluid is not bile-stained. Again, choline, a product of nerve degeneration, may be detected clinically and physiologically in the blood in cases where there is active degeneration of the nervous system. Leucocytosis of the cerebro-spinal fluid is one of the earliest signs of an organic disease of the nervous system. The acute diseases are associated with polynuclears, the chronic with lymphocytes. In diseases of the nervous system in which also the membranes are affected the mononuclears are in evidence, as in meningo-myelitis, general paralysis, and tabes. In functional neuroses, psychoses, and neuritis there is no lymphocytosis. It is probable that the leucocytosis is in response to the escape of products of degeneration into the cerebro-spinal fluid. Red blood corpuscles are not found in the cerebro-spinal fluid except in cases of hæmorrhages. It is only under exceptional conditions that the cerebro-spinal fluid contains micro-organisms, even though they are abundant in the blood. They are present, however, in cerebro-spinal meningitis and in tuberculous meningitis. The path of entry is uncertain, but possibly from the naso-pharynx viâ the perineural and perivascular lymphatics. In sleeping sickness the trypanosoma is found in the cerebro-spinal fluid. The coincidence in distribution between the fly, glossina palpalis, the trypanosoma in the blood of men and animals, and the sleeping sickness, strongly supports the view that its epidemiology depends on the biting fly being able to carry the parasite for days in its stomach. In post-mortem examinations a chronic meningo-encephalo-myelitis is found, the leading characteristic of which is the filling of the perivascular canalicular systems with large and small mononuclear leucocytes. It is undetermined whether this change is due to the invasion of the subarachnoid space by the trypanosoma, or whether it leads to secondary infection by acting as a carrier of micro-organisms, or by destroying the impermeability of the subarachnoid sac and thus allowing invasion by organisms normally present in the body. Portuguese observers found a diplococcus in the cerebro-spinal fluid and in the nervous substance, and this has been confirmed by Mott and others. The characteristic symptoms of the disease are undoubtedly due to the cerebral anæmia caused by the compression of the small vessels, by the accumulation of lymphocytes in the perivascular spaces and the mechanical interference thus produced in the circulation.

**Total Transverse Lesions of the Spinal Cord.**—The view of Bastian that total transverse lesions of the cord cause total abolition of the deep reflexes has recently been confirmed by Collier[2] from the investigation of a large number of cases. He states that in total transverse lesions not only are the knee-jerks and other deep reflexes permanently abolished in the region supplied by the caudal segment of the divided spinal cord, but in addition the muscles waste and lose their faradic excitability, and the sphincters lose their tone, the only sign of self-action remaining in the isolated part of the spinal cord being the occasional presence of certain of the skin reflexes in much reduced degree. These phenomena occur in the absence of any recognisable structural change in the ventral horn cells, ventral roots, and peripheral nerves of the paralysed region. The muscular wasting affects all the voluntary muscles supplied below the lesion equally, and it may come to be as severe as that seen in anterior poliomyelitis. Collier also found that in early stages of the development of these phenomena long continued faradisation of the lower limbs may causes a return of the lost knee-jerks. This indicates that the lumbo-sacral centres in man have not sufficient self-action to maintain the muscular tone when severed from the higher centres, but that they may be temporarily roused by peripheral stimulation into such a higher state of activity as will allow the knee-jerk to be obtained. The skin reflexes appear to be invariably abolished in severe transverse lesions, with the exception of the plantar reflexes; where life is prolonged all signs of reflex action slowly disappear. As regards visceral reflexes, after the occurrence of a total transverse lesion of the cord there may be temporary retention and reflex evacuation; but soon these cease, the orifices become patulous, constant escape occurs, and the bladder slowly contracts so that lavage becomes difficult. Trophic changes are very little in evidence in such cases, and bed sores do not occur if the skin is properly attended to. It seems clear that complete physiological abrogation of the functions of a transverse area of the spinal cord may exist without actual death of the elements involved, but not for any length of time. In a case of compression paraplegia, therefore, the onset of the flaccid state is a sign which indicates that operative interference for the relief of pressure must not be delayed, if it is to be of any avail.

[1] Brit. Med. Jour., Dec. 10, 1904. [2] Brain, 1904.

*(To be continued.)*

